# Unveiling Tirzepatide's Therapeutic Spectrum: A Dual GIP/GLP-1 Agonist Targeting Metabolic, Neurological, and Cardiovascular Health

**DOI:** 10.1155/ije/2876156

**Published:** 2025-10-01

**Authors:** Joya Ghaleb, Katy Kaleen Khouzami, Nicolas Nassif, Philippe Attieh, Mohammad Feras Al Ajlani, Jana Bou Sleiman, Ali Khalouf, Frederic Harb, Sami Azar, Amjad Kanaan, Hilda E. Ghadieh

**Affiliations:** Department of Biomedical Sciences, Faculty of Medicine and Medical Sciences, University of Balamand, P.O. Box 100, Al-Koura, Tripoli, Lebanon

**Keywords:** glucagon-like peptide-1, neuroprotection, nonalcoholic fatty liver disease, nonalcoholic steatohepatitis, obesity, tirzepatide, Type 2 diabetes

## Abstract

Tirzepatide, a novel dual glucose-dependent insulinotropic polypeptide (GIP) and glucagon-like peptide-1 (GLP-1) receptor agonist, has emerged as a groundbreaking treatment for Type 2 diabetes mellitus (T2DM) and obesity. Initially developed for glycemic control, recent clinical and preclinical data reveal its broader therapeutic potential across a range of metabolic and systemic conditions. This review explores tirzepatide's mechanisms of action, clinical efficacy, and safety profile, with particular attention to its impact on T2DM, obesity, cardiovascular health, metabolic-associated fatty liver disease (MAFLD), chronic kidney disease (CKD), and neurological disorders such as Alzheimer's and Parkinson's diseases. By addressing multiple pathophysiological pathways, including insulin resistance, inflammation, and oxidative stress, Tirzepatide presents a unique opportunity to redefine treatment paradigms beyond glycemic management. Our review also synthesizes recent evidence on the efficacy and safety of tirzepatide for obesity management specifically in Asian populations; a group frequently underrepresented in global trials. This demographic focus introduces a valuable dimension to the existing body of knowledge. As ongoing trials continue to evaluate its long-term effects, tirzepatide stands at the forefront of a new era in integrated cardiometabolic and neuroprotective therapeutics.

## 1. Introduction

Labeled as a “twin epidemic” [1], diabetes and obesity often go hand in hand, threatening our world today [2] in more ways than one. Not only can this epidemic lead to disrupting comorbidities, but statistics show [3] that it also significantly increases mortality rates worldwide [4]. Both diabetes and obesity are multifactorial in etiology resulting from a complex interaction between hereditary, environmental, and lifestyle factors [5]. Obesity, as defined by the World Health Organization (WHO), is a medical condition marked by an excessive accumulation of body fat, often determined by a high body mass index (BMI). It is associated with increased risks of various diseases such as diabetes, heart disease, and certain cancers, requiring both individual and societal approaches for prevention and management. Diabetes, according to WHO, is a multifaceted metabolic condition marked by disruption in the body's management of glucose. In Type 1 diabetes, insufficient insulin production results from the immune system targeting and destroying the pancreatic beta cells, which are responsible for insulin secretion. In Type 2 diabetes mellitus (T2DM), cells exhibit reduced responsiveness to insulin, alongside inadequate insulin production, contributing to elevated blood sugar levels. Obesity is a major risk factor for T2DM [6, 7]. Tirzepatide was marketed as a drug for the management of T2DM. So far, it has demonstrated unparalleled efficacy in the treatment of T2DM accompanied with improved glycemic regulation [8, 9]. Furthermore, it has been proven to produce remarkable weight loss in obese people. This is partly due to tirzepatide's mechanism of action as a double agonist, permitting it to assert a more significant decrease in hyperglycemia as compared to glucagon-like peptide (GLP-1) agonists on their own [8]. This review aims to provide a comprehensive overview of tirzepatide's emerging therapeutic roles beyond glycemic control and weight loss, including its potential benefits in cardiovascular, hepatic, renal, and neurological health.

## 2. Role of Tirzepatide in T2DM and Obesity Management

### 2.1. Mechanism of Action

**Figure 1:**
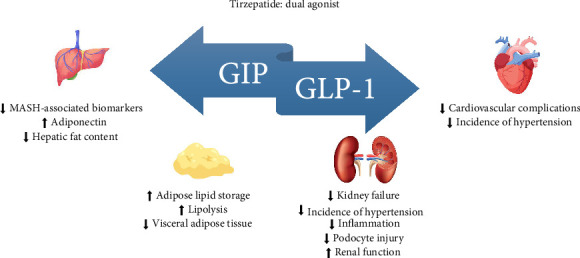
Tirzepatide effect on the kidney, adipose tissue, liver, and cardiovascular system.

Glucose-dependent insulinotropic polypeptide (GIP) and GLP-1 (Figure 1) are the two primary incretin hormones secreted from the intestine on ingestion of glucose or nutrients to stimulate insulin secretion from pancreatic β cells [10]. GIP and GLP-1 exert their effects by binding to their specific receptors, GIP receptor (GIPR) and the GLP-1 receptor (GLP-1R), both belonging to the G protein-coupled receptor family. Receptor binding increases the level of intracellular cyclic adenosine monophosphate (cAMP) in pancreatic β cells, thereby stimulating insulin secretion, glucose-dependently [7, 10]. In addition to their insulinotropic effects, GIP and GLP-1 play critical roles in various biological processes in different tissues and organs that express their receptors, including the pancreas, adipose tissue, bone, and the brain. Within the pancreas, GIP and GLP-1 together promote β-cell proliferation and inhibit apoptosis, thereby expanding pancreatic β-cell mass. In adipose tissues, GIP (but not GLP-1) facilitates fat deposition. In bone, GIP promotes bone formation, while GLP-1 inhibits bone reabsorption. In the brain, both GIP and GLP-1 are thought to be involved in memory formation as well as the control of appetite [10]. This multiorgan effect of tirzepatide prompts us to think about evaluating its potential beneficial effects beyond the management of diabetes and weight loss.

### 2.2. Administration

Currently, tirzepatide is only available subcutaneously through an injection [11]. The dosages available range from 2.5 mg/0.5 mL to 15 mg/0.5 mL. The prescribed dose is usually taken once weekly, and the initiation dose is 2.5 mg/0.5 mL. According to the variation in hemoglobin A1c (HbA1c) levels, changes in body weight, and side effects, prescribed doses are susceptible to an increase or a change. In patients prone to dehydration, tirzepatide should be used with caution due to its gastrointestinal side effects, particularly nausea, vomiting, and diarrhea which may increase the risk of volume depletion. This is especially important in elderly patients or those on diuretics, where dehydration could lead to acute kidney injury or hypotension [11]. In pregnant patients, there is inadequate information to evaluate for possible congenital disabilities in fetuses, but increased malformations have been observed in animal studies. Therefore, it is advisable for pregnant women to use tirzepatide only when the potential benefit justifies the potential risk imposed on the fetus. It is important to note that the efficacy of hormonal contraception, when used orally, decreases because of tirzepatide use. In lactating women on tirzepatide, there is no sufficient clinical data that support or argue against its use. However, due to its high molecular weight and large structure, it is theorized that the milk concentration of tirzepatide is less, and absorption is unlikely to occur due to the breastfed infant's gastrointestinal tract [11].

### 2.3. Monitoring

Typically, HbA1c and body weight should be measured during follow-up visits for patients on tirzepatide. For HbA1c, monitoring can be performed every 3 months. Because insulin sensitivity is highly improved for a patient on tirzepatide, they are instructed to more frequently monitor their blood sugar levels [11, 12]. Moreover, patients on tirzepatide should also be monitored for adverse effects, especially if an increased dose was prescribed. Since it is likely that there is an association between medullary thyroid carcinoma and tirzepatide, thyroid nodules are monitored during physical examinations. Their presence is coupled with serum calcitonin levels. If serum calcitonin levels exceed 50 ng/L, further assessment of the patient for medullary thyroid carcinoma is performed [11]. Finally, quality-of-life assessment for patients with T2DM is advised for an optimal standard of care.

### 2.4. Adverse Effects

While most users do not experience adverse effects, Tirzepatide may cause nausea, vomiting, constipation, and diarrhea, increasing in frequency with increasing doses [13]. Other than the gastrointestinal side effects [11], tirzepatide may rarely contribute to sinus tachycardia, acute kidney injury secondary to dehydration, pancreatitis, cholelithiasis, and cholecystitis.

### 2.5. Contraindications

It has been demonstrated that there is a possibility of developing medullary thyroid carcinoma in animal studies when on tirzepatide. Hence, tirzepatide is contraindicated in individuals with a personal or familial history of medullary thyroid carcinoma [11]. Tirzepatide is also contraindicated in individuals with a history of multiple endocrine neoplasia syndrome Type-2 (MEN 2). Moreover, users who have already experienced a hypersensitivity reaction to tirzepatide, have gallbladder disease, or suffer from diabetic retinopathy should avoid tirzepatide.

### 2.6. Toxicity, Efficacy, and Safety

Since tirzepatide has a relatively long half-life, patients who overdose on the medication should undergo prolonged monitoring. Currently, there is no known antidote to reverse tirzepatide overdose, but supportive care is indicated for control of symptoms [11]. According to Sinha et al., tirzepatide has better efficacy compared to many glucose-lowering medications such as dulaglutide, glargine, semaglutide, and insulin degludec and placebo drugs [14].

In the SURPASS program, a series of trials designed to investigate the efficacy and safety of tirzepatide, it was shown that the drug reduced HbA1C levels compared to the aforementioned drugs. As for weight outcomes, a significant weight loss was noted in all groups on tirzepatide in the SURPASS program. Moreover, tirzepatide leads to a reduction in liver fat, macroalbuminuria, blood pressure, and lipids [13]. SURPASS-4 evaluated the safety of tirzepatide in a high cardiovascular risk population with advanced T2DM for a duration of 2 years [14]. The assessment was performed using the major adverse cardiac event (MACE)–4 composite that includes cardiovascular death, myocardial infarction, stroke, and unstable angina hospitalization. These events were not increased in the groups receiving tirzepatide compared to the groups receiving glargine. Moreover, analysis of SURPASS-4 individuals showed that tirzepatide delayed the decline of estimated glomerular filtration rate (eGFR) and reduced the urine albumin to creatinine ratio (UACR) compared to insulin glargine. Eventually, individuals on tirzepatide had a low occurrence of end-stage kidney disease, death due to kidney failure, macroalbuminuria, and eGFR decline compared to individuals on insulin glargine [14].

As for its safety, only a few individuals reported serious side effects across all SURPASS groups. Mostly, these side effects were mild and dose-dependent. Serious hypoglycemia (blood glucose less than 54 mg/dL) in SURPASS-4 was reported less (9%) in individuals on tirzepatide than in individuals on insulin glargine (19%). However, in SURPASS-5, individuals on a background of insulin glargine and tirzepatide reported serious hypoglycemia (19%) more than individuals on a background of insulin glargine and placebo (13%) [14]. Therefore, unless combined with insulin or sulfonylurea, tirzepatide does not increase the risk of hypoglycemia.

### 2.7. Tirzepatide for Obesity Management in Asian Populations

Recent studies have begun to explore the efficacy and safety of tirzepatide specifically for obesity management in Asian populations. This novel theme is emerging as researchers investigate the drug's impact on weight reduction and its safety profile in diverse demographic groups. The focus on Asian populations addresses a critical gap in the literature and could lead to tailored therapeutic strategies for obesity management in these communities.

Kadowaki et al. conducted a randomized, double-blind, placebo-controlled Phase 3 trial that evaluated the efficacy and safety of once-weekly tirzepatide (10 mg or 15 mg) in Japanese adults with obesity (BMI ≥ 27 kg/m^2^ with ≥ 2 obesity-related conditions or ≥ 35 kg/m^2^ with ≥ 1, excluding diabetes). A total of 225 participants were included in the modified intention-to-treat population and assigned to tirzepatide 10 mg (*n* = 73), 15 mg (*n* = 77), or placebo (*n* = 75), alongside lifestyle modifications. At Week 72, mean bodyweight reduction was significantly greater with tirzepatide (−16.1% for 10 mg and −21.1% for 15 mg) compared to placebo (*p* < 0.0001). In addition, 94% (10 mg) and 96% (15 mg) achieved ≥ 5% weight reduction versus 20% with placebo. Improvements were also seen in cardiometabolic and body composition indices. Gastrointestinal adverse events (AEs) were more frequent in the tirzepatide groups but rarely led to discontinuation. Tirzepatide was effective and well-tolerated, offering a clinically meaningful weight loss in Japanese patients with obesity [15].

Zhao et al. conducted a randomized, double-blind, placebo-controlled Phase 3 trial to assess the efficacy and safety of tirzepatide in Chinese adults with obesity or overweight and at least one weight-related comorbidity, excluding diabetes. Conducted across 29 centers in China from September 2021 to December 2022, 210 participants were randomly assigned to receive tirzepatide 10 mg (*n* = 70), 15 mg (*n* = 71), or placebo (*n* = 69) once weekly for 52 weeks, alongside lifestyle intervention. At Week 52, mean body weight reductions were −13.6% and −17.5% with tirzepatide 10 mg and 15 mg, respectively, compared to −2.3% with placebo (both *p* < 0.001). In addition, ≥ 5% weight loss was achieved by 87.7% (10 mg) and 85.8% (15 mg) of participants versus 29.3% in the placebo group (*p* < 0.001). Gastrointestinal symptoms were the most common AEs, mostly mild to moderate, with few discontinuations (< 5%). Tirzepatide demonstrated significant weight loss benefits and a favorable safety profile in Chinese adults with obesity or overweight [16]. Furthermore, another study also assessed the safety profile of tirzepatide using data from the FDA AE Reporting System (FAERS) between Q2 2022 and Q4 2023. A total of 638,153 AEs were reported, with 8096 attributed to tirzepatide. Using the reporting odds ratio (ROR) method, 98 preferred terms showed statistically significant associations with tirzepatide (95% CI lower limit > 1). Common expected AEs included injection site pain, nausea, hemorrhage, diarrhea, and vomiting. Notably, unexpected AEs such as incorrect dosing, off-label use, extra doses, inappropriate administration schedules, and elevated blood glucose levels were also frequently reported. These findings highlight potential novel AE signals and underscore the value of real-world data in uncovering the broader safety profile of new medications. This study supports the ongoing monitoring of tirzepatide to ensure its safe use in clinical settings [17].

## 3. The Effects of Tirzepatide on Cardiovascular Disease

Due to tirzepatide's effect on the GLP-1R, one could expect similar protective benefits on cardiovascular health as other selective GLP-1R agonists [13]. Markers of cardiovascular health and disease were assessed in patients treated with tirzepatide. In the SURPASS-2 study conducted on T2DM, when comparing the effects of tirzepatide to a selective GLP-1R agonist, serum triglyceride and apolipoprotein C-III levels were significantly more reduced by tirzepatide than by both dulaglutide and placebo [18]. Tirzepatide treatment was also shown to produce a marked decrease in the levels of large and small triglyceride-rich lipoproteins such as diacylglycerol, phosphatidylethanolamines, and phosphatidylcholines [19]. However, a similar level of reduction in low-density lipoprotein (LDL) cholesterol and apolipoprotein B was seen with both tirzepatide and dulaglutide [18]. Moreover, tirzepatide therapy produced an increase in HDL cholesterol and lipoprotein lipase levels when compared to selective GLP-1 agonist treatment alone [20, 21]. Thus, from the preliminary evidence found from the previous trial, tirzepatide will have a positive impact on overall cardiovascular health [13].

### 3.1. The Impact of Tirzepatide on Aortic Inflammation and Atherosclerosis Development

To further strengthen the positive impact tirzepatide has on the heart, namely, atherosclerosis reduction [22], a study was conducted on mice involving GIPR and its impact on the cardiovascular system [23]. In human beings, GIPR is a receptor found to be expressed in the endothelial cells of some blood vessels, the pancreas, as well as in the heart [24]. In response to food intake, GIP binds to GIPR on beta cells of the pancreas, stimulating insulin secretion. To assess atherosclerosis development, researchers compared GIPR-positive mice to GIPR-knocked-out mice. An obvious increase in risk of aortic inflammation and atherosclerosis was seen in GIP- knocked out mice, despite maintained glucose homeostasis and reduced weight gain. [23]. This finding broadens the understanding of GIPR's role in mitigating inflammation-related pathophysiology, extending beyond its traditional function as an incretin in metabolic regulation.

### 3.2. Evaluating the Cardiovascular Safety and Efficacy of Tirzepatide in Patients With T2DM

In addition, the direct impact of tirzepatide on the incidence of cardiovascular complications has been evaluated. In a study published in The Lancet aiming to assess the efficacy and cardiovascular safety of tirzepatide compared to insulin glargine [25], participants from 14 different countries and all 5 continents were selected to participate in the Phase 3 study. All participants were T2DM patients being treated with a combination of metformin, sodium-glucose co-transporter-2, or sulfonylurea. They all had a high HbA1c level ranging between 7.5% and 10.5% and had a BMI of 25 kg/m^2 or higher. Most importantly, they all suffered from a cardiovascular disease or were indicated as high risk for the occurrence of cardiovascular events. This study occurred over a period of a minimum of 52 weeks, collecting and assessing MACEs. Participants were randomly assigned to groups either receiving a once-per-week subcutaneous injection of tirzepatide(5 mg, 10 mg, or 15 mg) or insulin glargine (100 U/mL). In addition to greatly reducing HbA1c levels, tirzepatide treatment was not associated with increased cardiovascular risk [7, 26].

Moreover, in the SURPASS-4 trial, subjects at high cardiovascular risk, including (but not limited to) those with coronary artery disease, cerebrovascular disease, or congestive heart failure, were assessed for the occurrence of MACE. It was found that for subjects treated with its highest dose, the cardiovascular risk of tirzepatide treatment was a low risk of 0.5, with only 11 MACE being recorded as compared to 62 MACE with insulin glargine [26]. This set of data is adequate as baseline evidence for the cardiovascular safety of tirzepatide, but a specific and longer clinical trial (SURPASS CVOT) is currently being conducted to give definite answers on the possible cardiovascular protective factor of tirzepatide [26].

### 3.3. The Role of Tirzepatide in Preventing Diabetic Heart Failure

In the assessment of the effects of tirzepatide on diabetic heart failure, various mechanisms are involved in the development of this disease [27]. Such mechanisms involve ischemic coronary artery disease, hypertension, or valvular disease, leading to cardiac hypertrophy and fibrosis. In a study conducted on diabetic mice, the effects of GIP on cardiomyocytes were studied [28]. Typically, myocardial transforming growth factor (TGF)–beta expression is upregulated in animal models with cardiac hypertrophy [29]. As such, the expression of both TGF-Beta 2 and Beta-MHC, another marker for cardiomyocyte hypertrophy, along with left ventricular (LV) wall thickness, cardiomyocyte size, and the presence of fibrosis were all measured in diabetic mice before and after GIP treatment for 6 weeks [28]. Pretreatment, diabetic mice exhibited a series of findings concomitant with heart failure such as a large LV wall thickness, a large cardiomyocyte size, a sizeable fibrotic area, and high levels of both TGF-Beta 2 and Beta-MHC. Posttreatment, all these manifestations were reduced. This suggests the effectiveness of tirzepatide, a GIPR agonist, in diabetic heart failure. Furthermore, upon extensive evaluation of the downstream signaling of TGF-Beta 2, it was found that compared to nondiabetic mice, diabetic mice have increased expression of certain cardiac proteins, such as p-p38, a protein kinase activated by genotoxic stress [30], and even gene expression, such as connective tissue growth factor (CTGF), or CTGF which is involved in multiple pathologies [31]. Both marker expressions were prevented with GIP treatment [28]. This extends the potential role of tirzepatide as not only a remedy but also as a possible preventative of diabetic heart failure. In addition, high glucose levels significantly increase nicotinamide adenine dinucleotide phosphate (NADPH) oxidase–driven superoxide levels, contributing to the progression of heart failure [32]. Treatment of diabetic mice with GIP agonists decreased NADPH oxidase–driven superoxide levels [28]. Through its various mechanisms of action, tirzepatide is a perfect candidate drug for both the treatment and prevention of diabetic cardiomyopathy.

### 3.4. The Therapeutic Effect of Tirzepatide in Obesity-Related Heart Failure

The cardiac magnetic resonance (CMR) substudy of the SUMMIT trial evaluated the effects of tirzepatide on cardiac structure and function in patients with obesity-related heart failure with preserved ejection fraction (HFpEF). The hypothesis was that tirzepatide would reduce LV mass and epicardial adipose tissue (EAT) in these patients. A total of 175 participants with obesity-related HFpEF were randomly assigned to receive either tirzepatide (2.5 mg–15 mg weekly) or a matching placebo. CMR imaging was conducted at baseline and 52 weeks. Of these, 106 patients completed the CMR with adequate image quality for analysis. The primary endpoint was the change in LV mass. The results showed that LV mass decreased by 11 g in the tirzepatide group compared to the placebo group (*p*=0.004). Paracardiac adipose tissue (EAT and pericardial fat) decreased by 45 mL in the tirzepatide group (*p* < 0.001). Changes in LV mass correlated with weight loss, waist circumference, and blood pressure reductions. In addition, changes in LV mass were associated with improvements in LV end-diastolic volume and left atrial volume [33]. In conclusion, tirzepatide treatment in patients with obesity-related HFpEF led to reductions in LV mass and paracardiac adipose tissue, and these changes were linked to weight loss. These findings suggest that tirzepatide could help reduce heart failure events in this population.

### 3.5. The Therapeutic Potential of Tirzepatide in Sepsis-Induced Cardiomyopathy and Ventricular Arrhythmias

Studies have shown that there is a potential therapeutic role for tirzepatide on sepsis-induced cardiomyopathy and sepsis-induced ventricular arrhythmias [34]. Cardiomyopathy is one of the potential complications of sepsis, conveyed by a reversible LV systolic dysfunction. In fact, the problem does not lie in the complication itself but rather in the treatment, since options are very limited. Liu et al. have discussed in their study the function of tirzepatide in the treatment of the sepsis-induced cardiomyopathy. The effects of tirzepatide on sepsis-induced cardiomyopathy were studied in lipopolysaccharide (LPS)-treated mice. LPS is used to induce an inflammatory response in the mice, activating macrophages, and therefore providing the model of sepsis-induced cardiomyopathy in the studied mice. Survival rate was studied at first by comparing the effects on LPS-treated mice with and without the administration of tirzepatide [34]. Results have shown a decrease in mortality rate caused by sepsis-induced cardiomyopathy in mice that were treated with tirzepatide, although, these results did not reach statistical significance. In addition, decreased serum levels of creatine kinase-MB (CK-MB), lactate dehydrogenase (LDH), and aspartate transferase (AST) were found in mice that were treated with tirzepatide. It was also found that levels of some cardiac proteins, such as tumor necrosis factor alpha (TNF-α), interleukin (IL)-6, and IL-1β, also significantly decreased. The study by Liu et al. also examines the effect of tirzepatide on the toll-like receptor 4/nuclear factor beta/NOD-like receptor pyrin domain containing 3 (TLR4/NF-kB/NLRP3) pathway [34]. The TLR4/NF-kB/NLRP3 is a pathway that plays a vital role in apoptosis and the inflammatory process induced by LPS as a result of sepsis [35]. LPS usually elevates the expression levels of proteins TLR4, p-NF-kB, and NLRP3 [34]. However, the results of this experiment have shown a recovery of TLR4, p-NF-kB, and NLRP3 protein levels to normal ranges in the mice treated with tirzepatide. It was therefore concluded that tirzepatide inhibits the activation of the TLR4/NF-kB/NLRP3 inflammasome in the hearts of mice that were administered LPS.

Lastly studied by Liu et al. is the effect of tirzepatide on ventricular arrhythmia triggered by sepsis. The experiment conducted shows an increase in QRS intervals and corrected QT (cQT) intervals in mice that were administered LPS. In contrast, in mice treated with tirzepatide, there was a significant attenuation in both the QRS and cQT intervals [34].

This mechanism behind the decrease in mortality and morbidity in tirzepatide-treated mice can be explained by the fact that tirzepatide attenuates the excessive inflammatory response induced by LPS which mimics the physiological effects of sepsis on mice [34]. In conclusion, tirzepatide leads to improvement in terms of survival and cardiac function caused by sepsis-induced cardiomyopathy and may serve as a future potential agent for those with sepsis-induced cardiomyopathy.

### 3.6. The Role of Tirzepatide in Blood Pressure Reduction

To assess the effectiveness of tirzepatide in blood pressure control, the SURMOUNT-1 trial was conducted in individuals with obesity or overweight, focusing on the relationship between weight loss and hypertension. After 72 weeks of tirzepatide treatment, participants experienced significant reductions in both systolic and diastolic blood pressure. The treatment led to a rapid decline in blood pressure over the first 24 weeks, followed by stabilization, resulting in a net reduction of 6.8 mm Hg in systolic and 4.2 mm Hg in diastolic pressure compared to placebo [36]. At Week 72, participants on tirzepatide were more likely to have normal blood pressure (58.0%) compared to those on placebo (35.2%). These effects were consistent across various subgroups, including baseline blood pressure categories. Mediation analysis revealed that weight loss accounted for 68% of the systolic and 71% of the diastolic blood pressure reduction. Although AEs related to low blood pressure were rare, they occurred more frequently in the tirzepatide group [36]. Overall, tirzepatide was shown to reduce blood pressure in individuals with obesity or overweight, primarily through weight loss, with minimal AEs related to low blood pressure.

## 4. The Effects of Tirzepatide on Liver Disease

In the liver, the balance of lipid metabolism is between fatty acid (FA) input and output. FA input is due to free FAs (FFAs) being esterified to triglycerides. Free FA are either released from subcutaneous adipose tissue or through de novo lipogenesis. FA output is due to FA oxidation, when FAs are being broken down for energy. Another way is using very LDL (VLDL) for their export. If this balance is shifted where FA input exceeds output, steatosis takes place [37]. MASLD can present as steatosis which follows a benign, non-progressive course. A more concerning presentation is metabolic dysfunction-associated steatohepatitis (MASH). MASH is characterized by liver cell injury and inflammation. Moreover, it can lead to liver cirrhosis and hepatocellular carcinoma [38].

### 4.1. The Pathogenesis of Metabolic-Associated Fatty Liver Disease (MAFLD) and MASH

MAFLD and its more severe form, MASH, involve a complex interplay of multiple factors, including abdominal obesity, T2DM, and hyperlipidemia. A meta-analysis found that 55% of T2DM patients have MAFLD, and 35% have MASH [39]. Obesity, defined as a BMI > 30, is a significant contributor, with up to 80% of obese individuals developing MAFLD [37]. The multiple-hit theory explains the development of MAFLD in genetically susceptible individuals. Insulin resistance, nutritional factors, hormones from adipose tissue, gut flora, and genetic and epigenetic factors all contribute to the diseased liver phenotype [40]. Impaired insulin sensitivity promotes adipose tissue hydrolysis, increasing circulating FFAs, which accumulate in hepatocytes, leading to steatosis (the first hit) [41]. The accumulation of triglycerides in hepatocytes results in excessive FA oxidation, creating oxidative stress, lipotoxicity, and inflammation (the second hit), contributing to liver fibrosis and cirrhosis [40]. Genetic factors, such as nonsynonymous single-nucleotide polymorphisms (SNPs) in the patatin-like phospholipase domain–containing protein 3 (PNPLA3) gene, further influence the severity of liver damage. These alleles are linked to increased steatosis, MASH, and a higher risk of hepatocellular carcinoma [42]. These genetic variations play a crucial role in regulating hepatic fat levels, making them key factors in the progression from MAFLD to MASH and potentially liver cancer.

### 4.2. Potential Role of Tirzepatide in the Treatment of MASH

Although maintaining an adequate body weight, eating healthy, and exercising regularly is the best treatment for MAFLD, more research on tirzepatide has shown it to be an effective treatment for MASH patients [43]. Tirzepatide causes an increase in adiponectin, and according to a study performed by Shabalala et al., adiponectin is inversely related to lipid accumulation [44]. This is why new treatments target adiponectin in order to decrease lipid buildup in the liver. A study performed by Hartman et al. showed that T2DM patients being treated with tirzepatide revealed lower MASH-related biomarkers and an increase in adiponectin, a cytokine beneficial in the regulation of glucose levels, lipid metabolism, and insulin sensitivity [10, 45, 46]. In the post hoc analyses of the study, a decrease in both serum AST and serum alanine aminotransferase (ALT) levels was recorded [47].

The majority of the incretin impact is attributed to GLP-1 and GIP together, a physiological mechanism that developed to increase insulin production after meal absorption [48]. These receptors, expressed on pancreatic β cells, when activated, augment insulin secretion [49]. An agonist that has an affinity to both GLP-1R and GIPR is more potent in decreasing blood glucose levels along with body weight than either selective agonist [50]. In rodents, recent studies have found that GIPRs are found on the arcuate nucleus of the hypothalamus. Upon stimulation, these receptors result in decreased food intake and body weight. The effect of this stimulation is more potent when GIP is co-administered [51].

Patients suffering from MASH will have high levels of Keratin-18 (K-18), which is a marker for the diagnosis of MASH in their serum due to hepatocyte apoptosis that cleaves K-18. Giving higher doses of tirzepatide (10 and 15 mg) lowers ALT, thus lower levels of procollagen III (pro-C3), which is a fibrosis marker in T2DM patients, and K-18 were seen [47]. The results of the study supported this, showing that 15 mg of tirzepatide caused a decrease in pro-C3 by Week 26. Furthermore, it was seen that higher doses of tirzepatide further lowered biomarkers for MASH. However, due to limitations in the study (defective data and patients unequally distributed into groups), these results cannot be used as evidence of the consequences of tirzepatide on MASH. More research is needed to support these results [47].

### 4.3. Impact of Tirzepatide on Adipose Tissue Metabolism and Fatty Liver in Patients With T2DM and Obesity

Patients suffering from T2DM and obesity have lower adipose tissue blood flow (ATBF) because these patients experience an increase in fatty tissue mass; however, the increase is not reciprocated in the blood supply [52]. This may explain how a GIP agonist helps in adipose tissue lipid storage and adipocyte metabolism (lipolysis), which in turn leads to less fat deposits in the liver [50]. According to a subanalysis study of the SURPASS-3 trial, which comparedtirzepatide to insulin degludec, it showed that tirzepatide was associated with a more noticeable reduction in visceral adipose tissue (VAT) and liver fat content [53]. Tirzepatide exhibits a dual effect given that it is a dual agonist: GLP-1 leads to the postprandial secretion of insulin and inhibits glucagon. Moreover, GLP-1 increases satiety in two ways: centrally (via the hypothalamus) and peripherally (by slowing down gastric emptying). The second agonist, GIP, has a complex effect on glucagon but it was shown to increase insulin sensitivity by decreasing ectopic intramuscular deposition of fat and increasing fat storage in white adipose tissue [54]. Insulin sensitivity is important because of the inhibitory effect of insulin on lipolysis. Decreased lipolysis causes an increase in FFAs in the liver thus increasing FA input [55]. As explained earlier, by decreasing the VAT and fat content in the liver, the FA input would decrease, leading to a better control over fat content, glucose, and weight.

## 5. The Effects of Tirzepatide on Kidney Disease

As tirzepatide's role has been widely centered around decreasing the incidence of complications of diabetes mellitus, one must consider its impact on the development of diabetic nephropathy and chronic kidney disease (CKD). It is hypothesized that the podocyte injury seen in such conditions is largely due to underlying inflammatory conditions [56].

### 5.1. Reactive Oxygen Species (ROS) and Advanced Oxidation Protein Products (AOPPs) in Podocyte Damage and Diabetic Nephropathy Pathogenesis

Experimental studies have shown a direct link between increased ROS generation and podocyte damage. For example, a study by Gwinner et al. demonstrated that a transient ROS surge induced glomerulonephritis, causing podocyte defects, structural damage, and DNA injury through ROS interactions with intracellular molecules [57]. A key marker of oxidative stress is AOPPs, plasma byproducts of oxidative stress [58], which are elevated in diabetic patients and contribute to diabetic nephropathy by increasing proinflammatory molecules such as NADPH oxidase, NF-κB, and ROS [56]. Chronic accumulation of AOPPs is linked to glomerulosclerosis and podocyte injury, partly via the activation of the apoptotic pathway involving caspase-3 through superoxide generation by NADPH oxidase [59]. AOPPs also stimulate ROS production in podocytes, potentially through proteins such as CCAAT/enhancer binding protein (C/EBP) and glucose-regulated protein 78 (GRP78), resulting in podocyte apoptosis [58]. In addition, AOPPs upregulate the Wnt/β-catenin pathway through the receptor for advanced glycation end-products (RAGE) receptor, contributing to podocyte dysfunction [60]. Wnt/β-catenin activation promotes Snail1 production, which inhibits nephrin expression [61], essential for glomerular filtration, and leads to the epithelial–mesenchymal transition (EMT) [62]. This pathway also downregulates Wilms tumor protein expression, a key factor for podocyte protection, contributing to proteinuria [63]. Furthermore, the Wnt/β-catenin pathway and the renin-angiotensin system (RAS), which generates ROS [64], create a cycle of ongoing glomerular damage [65].

### 5.2. Exploring the Nephroprotective Potential of Tirzepatide: Insights From GIP and GLP-1 Agonism in Diabetic Nephropathy

#### 5.2.1. GIP Agonism Effect in Diabetic Nephropathy

**Figure 2:**
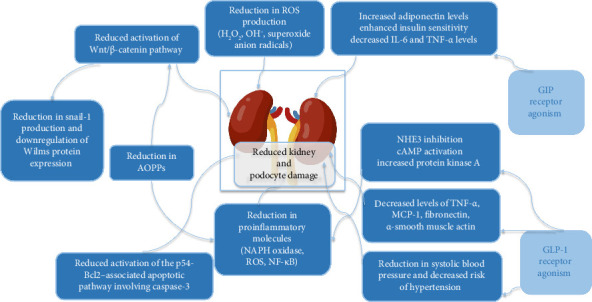
GLP-1 and GIP agonism effects on kidney risk factors and inflammatory and biochemical–mediated damage of podocytes.

The nephroprotective effect of tirzepatide has not been directly studied, however, the effect of GIP agonism on inflammation has been extensively documented (Figure 2). It has shown a pronounced anti-inflammatory effect, leading to decreased levels of various proinflammatory cytokines, decreased oxidative stress, as well as increased levels of adiponectin, which exerts an anti-inflammatory effect, as well as increases insulin sensitivity [66]. AdipoRon, an adiponectin receptor agonist, confirms the favorable effect on diabetic nephropathy, as it was shown to attenuate the hypertrophic glomerular response [66]. As for the decrease in levels of proinflammatory cytokines such as IL-6, IL-1β, and TNF-α [67], their effect has been studied in regards to kidney injury. First, it was recorded that blocking TNF-α in rats with diabetic nephropathy leads to marked amelioration in kidney function markers, decreased inflammation, and cell death [68]. Furthermore, increased TNF-α levels have been recorded in studies evaluating end-stage renal disease patients and were found to be directly correlated with increased albuminuria levels and inversely correlated with GFR levels [69]; suggesting its detrimental effect on kidney health. Lastly, a study evaluating the impact of IL-1β gene polymorphism, leading to increased IL-1β levels, was shown to correlate with an increased rate of kidney disease in T2DM, mainly diabetic nephropathy [8]. Thus, it can be inferred that a decrease in such inflammatory markers may be beneficial in preventing kidney disease and maintaining kidney function.

#### 5.2.2. GLP-1 Agonism Effect in Diabetic Nephropathy

As for GLP-1 (Figure 2), it has a more direct role in decreasing kidney oxidative stress, due to the presence of receptors in proximal tubular (PT) cells and kidney smooth muscle. In the renal PT cells, GLP-1 activation leads to sodium–hydrogen exchanger 3 (NHE3) inhibition, causing diuresis and natriuresis, possibly improving tubuloglomerular feedback [70]. On vascular and capillary walls, GLP-1 activation leads to cAMP activation, which then increases protein kinase A activity. This leads to a marked decrease in oxidative stress due to its role in inhibiting NADPH oxidase, leading to decreased superoxide generation, and thus downregulating one of the major causes of diabetic nephropathy [71]. It was also found that GLP-1R agonists decrease many proinflammatory biomarkers usually responsible for the development of diabetic nephropathy such as TNF-alpha, fibronectin, monocyte chemoattractant protein-1 (MCP-1) and alpha-smooth muscle actin [72]. Two GLP-1R agonists, liraglutide and exendin-4, have been shown to decrease TGF-β1, NF-κB, and intercellular adhesion molecule-1 (ICAM-1) levels, leading to reduced oxidative stress [72]. GLP-1Rs are also present in juxtaglomerular cells that secrete renin; thus, their activation may lead to various nephroprotective effects. They include improved glomerular function, decreased renal atherosclerosis rates, increased natriuresis, decreased endothelin-dependent vasodilation, and an improvement in tubuloglomerular feedback [73].

In addition to the direct nephroprotective effect, GLP-1R agonists have indirect protective effects as they decrease the incidence of many risk factors associated with kidney injury. Besides decreased body weight and glycemia, a decrease in systolic blood pressure by 2.22 mmHg was noted across 33 clinical trials [74], thus decreasing the incidence of hypertension, one of the main risk factors involved in the development of diabetic nephropathy [75]. GLP-1R agonist treatment also leads to a mild decrease in cholesterol levels (total, LDL, and triglyceride), an additional factor that participates in the pathogenesis and progression of such kidney disease [76]. Thus, both GIP and GLP-1 agonists have independently proven themselves to be efficient in protecting the kidneys. A post hoc analysis conducted by Apperlo et al. showed the potential nephroprotective effect of tirzepatide due to its dual agonist effect. This analysis aimed to evaluate the effects of tirzepatide (5, 10, and 15 mg) on UACR compared to active and placebo treatments in a broad population from the SURPASS-1–5 trials, including individuals with CKD. The results showed that tirzepatide significantly reduced UACR at Week 40/42 compared to pooled comparators, with the following reductions: −19.3% for 5 mg, −22.0% for 10 mg, and −26.3% for 15 mg. The reduction in UACR was most pronounced in participants with baseline UACR ≥ 30 mg/g. Mediation analysis suggested that approximately 50% of the reduction in albuminuria was related to weight loss. However, no significant difference in eGFR was observed between tirzepatide and comparators. These findings suggest that tirzepatide is associated with a clinically relevant decrease in UACR, indicating a potential kidney-protective effect in people with T2DM, including those with CKD [77].

## 6. Neuroprotective Effects of Tirzepatide

Recent preclinical studies demonstrate the beneficial effects of tirzepatide on the nervous system [78], particularly in the settings of both Alzheimer's and Parkinson's disease (PD) [79].

### 6.1. Neuroprotective Effects of Tirzepatide on PD

PD is a prevalent neurodegenerative disorder characterized by motor symptoms such as tremors, stiffness, slowness of movement, and balance problems [80]. These symptoms result from the progressive loss of dopamine-producing neurons in the brain. While the exact cause of PD remains unknown, research has identified several genetic factors and environmental influences that may contribute to its development. The hallmark of PD is the loss of dopaminergic neurons in the substantia nigra pars compacta (SNpc), leading to diminished dopamine levels in the striatum. Microscopically, the pathological hallmark of PD is the presence of abnormal cytoplasmic deposits within neuronal cell bodies, known as Lewy bodies (LBs), which are composed of the protein α-synuclein. These protein aggregates disrupt normal brain function and contribute to the disease's progression. Several mechanisms are implicated in PD pathogenesis, with α-synuclein aggregation being central to the development of the disease. Abnormal protein clearance, mitochondrial dysfunction, and neuroinflammation also play important roles in the onset and progression of PD [80]. For this reason, GLP-1R agonists have been considered in the setting of PD. GLP-1 RAs have been shown to enhance mitochondrial biogenesis and function, leading to reduced oxidative stress [78].

Activation of the GLP-1R stimulates the PI3K/Akt/mTOR pathway, which in turn upregulates the expression of antioxidant enzymes and reduces ROS production. In addition, GLP-1 RAs increase the levels of glutathione, a key antioxidant, by stimulating the transcription of glutamate–cysteine ligase, the rate-limiting enzyme in its synthesis. These effects collectively mitigate oxidative damage and support neuronal survival, potentially having a beneficial role in PD pathogenesis [78]. In addition, to help decrease neuroinflammation, a key factor driving PD pathogenesis, GLP-1 RAs modulate immune cell activity and suppress the release of proinflammatory cytokines, such as TNF-α, IL-6, and IL-1β, all of which play a major role in neuroinflammation. These cytokines also inhibit the NF-κB pathway, a mechanism central to the regulation of inflammatory responses. By dampening inflammation and modulating microglial activity, GLP-1 RAs create a neuroprotective environment that supports neuronal survival [78]. Following on that note, GLP-1 RAs exhibit strong antiapoptotic effects by activating the PI3K/Akt pathway, promoting cell survival by inhibiting proapoptotic factors such as Bax and caspase-3 and upregulating antiapoptotic proteins such as Bcl-2. They also protect against mitochondrial dysfunction, preventing the release of Cytochrome c and stabilizing mitochondrial membranes, thereby inhibiting apoptosis. This mitochondrial protection is especially critical in PD, where neuronal loss is driven by apoptotic processes [78]. Moreover, GLP-1 RAs have been shown to reduce the accumulation of α-synuclein and its phosphorylated forms, thereby mitigating their toxic effects [78].

These theories all come into play in some of the preclinical studies of GLP-1 RAs in animals, where it was demonstrated that GLP-1 RAs can reduce dopaminergic neuron loss and improve motor function. Moreover, an open-label trial tested exenatide, a GLP-1 RA, as a potential treatment for PD in a no-randomized, open-label study with 45 patients. 21 patients received subcutaneous exenatide injections, while 24 patients continued their conventional PD treatment. The trial duration was 12 months, with assessments conducted at baseline, 6 months, and 12 months. Motor and cognitive functions were evaluated using the Movement Disorders Society Unified PD Rating Scale (MDS-UPDRS). At the 12-month assessment, patients in the exenatide group exhibited a mean improvement of 2.7 points on the MDS-UPDRS, while the control group showed a mean decline of 2.2 points [81]. This difference was statistically significant (*p*=0.037). Nonmotor symptoms, including cognitive functions, also improved in the exenatide group. The treatment was well tolerated, with weight loss being a common side effect. No serious AEs were reported, followed by a 12-week washout period. The trial showed that patients treated with exenatide exhibited significant improvements in motor function (measured by the UPDRS motor score) that persisted even after stopping the drug. The study also indicated potential neuroprotective effects, with no significant progression of PD during the trial [81].

### 6.2. Neuroprotective Effects of Tirzepatide on AD

AD is a progressive neurodegenerative disorder characterized by cognitive decline, memory loss, and behavioral changes. The majority of AD cases are of late-onset, occurring after the age of 65, and are considered sporadic, with no clear genetic inheritance pattern. However, a small percentage of cases are familial, resulting from mutations in specific genes [82]. Genetic mutations play a significant role in the development of early-onset familial AD. Mutations in the amyloid precursor protein (APP) gene and presenilin genes (PSEN1 and PSEN2) are associated with the production of abnormal amyloid-beta (Aβ) peptides, leading to amyloid plaque formation in the brain. In late-onset AD, the apolipoprotein E (APOE) ε4 allele is a major genetic risk factor, influencing Aβ deposition and clearance. The accumulation of Aβ plaques and tau neurofibrillary tangles is a hallmark pathological feature of AD [82]. Aβ peptides aggregate to form plaques that disrupt neuronal communication and trigger inflammatory responses. Tau, a microtubule-associated protein, becomes hyperphosphorylated and forms tangles inside neurons, impairing their function and leading to cell death. Moreover, both neuroinflammation [83] and oxidative stress play a role in the pathogenesis of AD [82].

Tirzepatide has been shown to mitigate these effects via different mechanisms [79, 84]. First, tirzepatide has been shown to increase the expression of brain-derived neurotrophic factor (BDNF) and phosphorylated cAMP response element–binding protein (pCREB) [79], both of which are critical for neuronal growth and survival. These effects may counteract the neurodegenerative processes observed in AD. In addition to that, tirzepatide influences the expression of microtubule-associated protein 2 (MAP2), growth-associated protein 43 (GAP43), and ATP/GTP binding protein-like 4 (AGBL4), which are involved in neuronal differentiation and synaptic plasticity. These changes may enhance neuronal connectivity and function. More importantly, tirzepatide influences the expression of microRNAs, such as miRNA-34a, miRNA-212, and miRNA-29c, which are involved in the regulation of neuronal growth, apoptosis, and differentiation, respectively [79, 84].

In a study performed on AD model mice, tirzepatide was administrated intraperitoneally on APP/PS1 mice for 8 weeks at a dose of 10 nmol/kg once weekly. Assessment parameters included GLP-1R expression, amyloid plaque density, neuroinflammatory markers, neuronal apoptosis, and markers of brain glucose metabolism. In addition, mitochondrial function and ROS production were evaluated. Tirzepatide administration led to a significant reduction in GLP-1R expression in the cortex and hippocampus, as evidenced by decreased phosphorylation of protein kinase A (PKA) substrates at Thr197. Moreover, Aβ plaque density was significantly reduced in the cortex of tirzepatide-treated mice. Astrocytic activation, indicated by glial fibrillary acidic protein(GFAP) expression, was also notably decreased in the cortex and hippocampus, suggesting a reduction in neuroinflammatory responses. However, neuronal ROS production was reduced, while astrocytic ROS levels increased, suggesting possible differential effects on mitochondrial function [85].

## 7. Conclusion

Tirzepatide, a dual GIP/GLP-1R agonist, has demonstrated remarkable efficacy in improving glycemic control and promoting weight loss in individuals with T2DM and obesity. Beyond these primary effects, growing evidence supports its potential benefits across cardiovascular, hepatic, renal, and neurological systems. These pleiotropic effects, largely mediated through enhanced insulin sensitivity, anti-inflammatory pathways, and metabolic regulation, position tirzepatide as a promising agent in the integrated management of chronic metabolic diseases. As clinical trials continue to evolve, tirzepatide may help redefine therapeutic strategies beyond conventional diabetes care.

## Data Availability

Data sharing is not applicable as no new data were generated.
